# Supplementary material for: Highly efficient field-free switching of perpendicular yttrium iron garnet with collinear spin current
Source: Nat Commun. 2024 Apr 13;15:3201. doi: 10.1038/s41467-024-47577-x (PMC11016059; doi:10.1038/s41467-024-47577-x)
Supplement: Supplementary file 1 — Supplementary Information [file 41467_2024_47577_MOESM1_ESM.pdf]

Supplementary Information

Highly efficient field-free switching of perpendicular yttrium iron garnet with collinear spin current

Man Yang[1,#], Liang Sun[1,#], Yulun Zeng[1], Jun Cheng[1], Kang He[1], Xi Yang[1], Ziqiang Wang[1], Longqian Yu[1], Heng Niu[1], Tongzhou Ji[1], Gong Chen[1], Bingfeng Miao[1,*], Xiangrong Wang[2,3,*], Haifeng Ding[1,*]

[1]*National Laboratory of Solid State Microstructures, Department of Physics, Nanjing University, and Collaborative Innovation Center of Advanced Microstructures, Nanjing 210093, P.R. China*

[2]*Physics Department, The Hongkong University of Science and Technology, Clear Water Bay, Kowloon, Hongkong*

[3]*PHKUST Shenzhen Research Institute, Shenzhen 518057, P.R. China*

[#]These authors contribute equally to this work

*Corresponding author: bfmiao@nju.edu.cn, phxwan@ust.hk, hfding@nju.edu.cn

Content

- **Supplementary Note 1. Material Selection**
- **Supplementary Note 2. The hysteresis loops for 30, 60, 90-nm YIG on NGG/Pt(0.5 nm)**
- **Supplementary Note 3. Discussion about the decouple layer**
- **Supplementary Note 4. The microscopic image of the thermoelectric measurement**
- **Supplementary Note 5. Correlation between the thermal voltage loop and the magnetization loop**
- **Supplementary Note 6. SOT-switching measurement of demagnetized Py**
- **Supplementary Note 7. The normalization procedure for P-MOKE measurement**
- **Supplementary Note 8. The coupling between Py and PMA-YIG**

Supplementary Note 1. Material Selection

We choose Py as the spin-charge interconversion layer based on two factors. First, Py has been reported to have considerable spin-charge conversion efficiency in several previous works[1-4]. It can be anticipated that the field-assisted switching current threshold from the spin Hall effect itself would not be too high. Second, Py is a magnetic material which may contain the anomalous spin Hall effect. The aid of anomalous spin Hall effect may further reduce the switching current threshold to a value smaller than the typical heavy metal Pt, as reported in our manuscript. A nonmagnetic layer, Ag, is used to magnetically decouple the PMA-YIG and Py, and ensure a high ratio of pure spin current can be transferred between them. Meanwhile, it also has a low spin-charge conversion efficiency[3], thus not complicating the important role of anomalous spin Hall effect in Py on the highly efficient field-free switching. We compared Cu and Ag, two typical materials with long spin diffusion length[5, 6] and found that Ag is a better choice as the spin current can be transferred more efficiently (Supplementary Fig. 1).

It was reported that the spin diffusion length of Ag and Py are 700 nm [6] and 2.5 nm[1], respectively. As we are focusing on the field-free switching and Ag will provide the shunting effect, we did not measure the spin diffusion length of Ag. Our SSE measurements show the inserting of 4-nm Ag almost does not influence the transport of pure spin current (Supplementary Fig. 1), indicating a long diffusion length of Ag. Our Py thickness dependent measurements yield the diffusion length of Py to be 4.2±1.8 nm, in good agreement with previous estimated value of 2.5 nm.

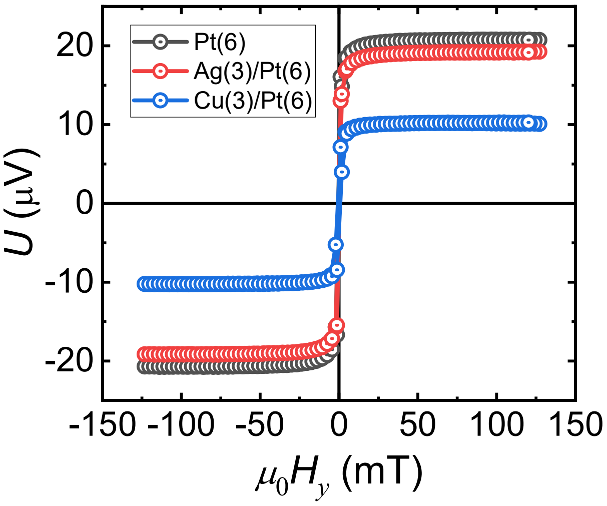

Supplementary Figure 1. The spin Seebeck effect signal measured in YIG/X(Ag, Cu)/Pt and YIG/Pt. The units of the numbers in parentheses are nm. The YIG film was deposited on thermally oxidized silicon substrates by RF magnetron sputtering and annealing at 800℃ for 1 hour in the atmosphere. Ag, Cu and Pt was deposited by DC magnetron sputtering. The heater is made from Ti(10 nm)/Cu(30 nm)/Ti(20 nm) and is insulated with the sample by 200-nm SiO_2 . An AC current with an amplitude of 100 mA and a frequency of 187 Hz is applied in the heater.

Supplementary Note 2. The hysteresis loops for 30, 60, 90-nm YIG on NGG/Pt(0.5 nm)

In this work, we find that only 10-nm YIG shows PMA, while 30, 60, 90-nm YIG show in-plane anisotropy (Supplementary Fig. 2). The in-plane coercive fields of 30, 60, 90-nm PMA-YIG are 3.8, 2.5, 2.3 mT, respectively. The thickness dependent spin reorientation transition is originated from the competition of the dipole interaction and the interfacial PMA caused by the out-of-plane compressive strain.

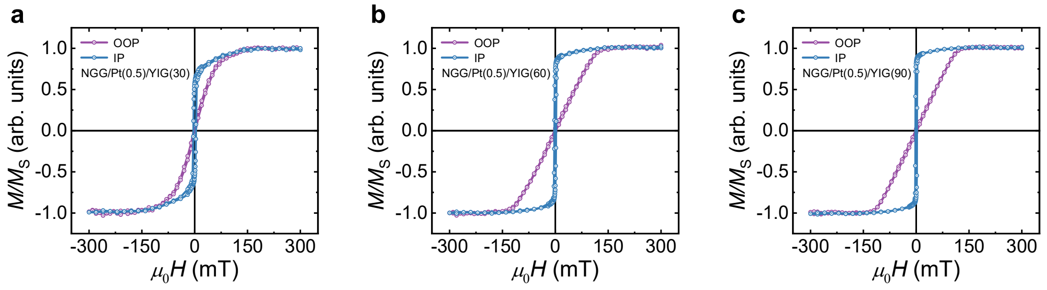

Supplementary Figure 2. Hysteresis loops of 30, 60, 90-nm YIG for **a**, **b** and **c**, respectively. Purple loops are the out-of-plane loops and blue loops are in-plane loops.

Supplementary Note 3. Discussion about the decouple layer

The non-magnetic metal, Ag is needed to reduce the magnetic coupling between PMA-YIG and Py. As shown in Supplementary Fig. 3, when Py is directly deposited on YIG (10 nm), the strong magnetic coupling between them changes the magnetic behavior of YIG and it no longer has large remnant magnetization along the perpendicular direction. After inserting 4-nm thick Ag, YIG film shows a square loop almost identical with the one without capping, indicating small coupling between YIG and Py.

Since Ag is also a material with high conductivity, it may increase the switching current threshold due to the shunting effect. In the future, Ag could be replaced with a material with high resistivity and high spin current conductivity. In such case, the switching current density could be further lowered. NiO could be a good candidate if the coupling between NiO and the other two ferromagnetic materials are weak.

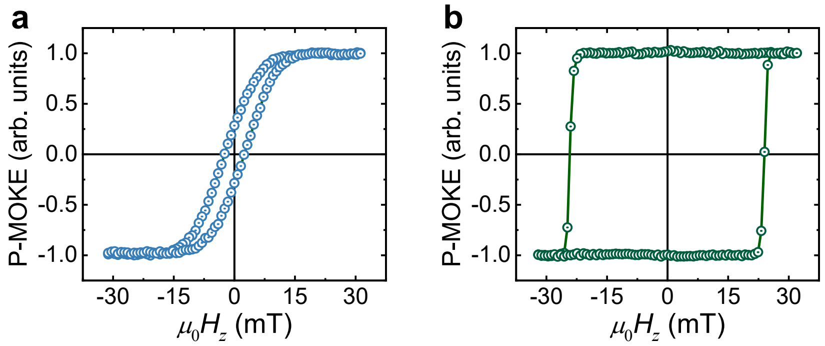

Supplementary Figure 3. a and **b** are the hysteresis loops of PMA-YIG(10 nm)/Py(6 nm)/SiO_2(6 nm) and PMA-YIG(10 nm)/Ag(4 nm)Py(6 nm)/SiO_2(6 nm) in out-of-plane measured by polar MOKE.

Supplementary Note 4. The microscopic image of the thermoelectric measurement

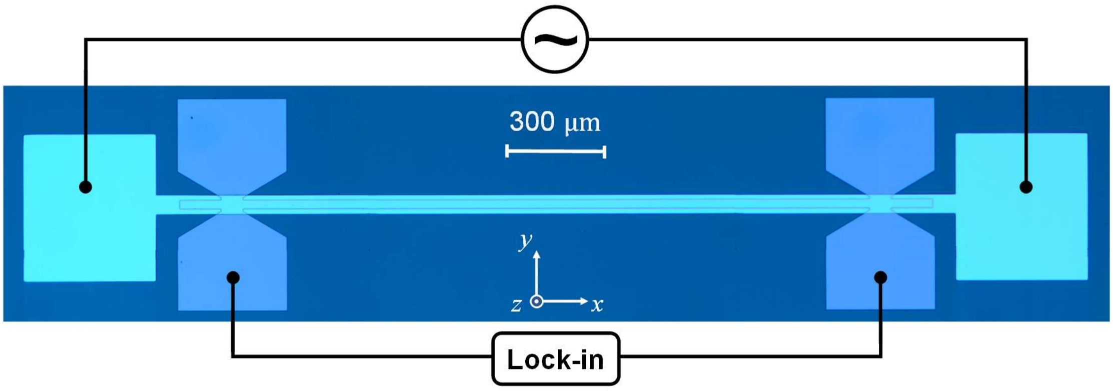

Supplementary Figure 4. The microscopic image of the thermoelectric measurement. The greenish blue, light blue and dark blue parts are the heater, sample and substrate, respectively.

Supplementary Note 5. Correlation between the thermal voltage loop and the magnetization loop

The thermal voltage loop (Fig. 2c in main text) has two origins. One is the anomalous Nernst effect (ANE) from Py, which scales with M_y of Py. The other one is the inverse spin Hall effect (ISHE) in Py, which scales with M_y of YIG. Generally, the scaling factors for ANE and ISHE with the magnetization are different. This results in a slight difference between the thermal voltage loop (Fig. 2c in main text) and the magnetization loop (Fig. 2d in main text). After the deconvolution of their individual contributions, one can find that they indeed have one-to-one correspondences (Supplementary Fig. 5.).

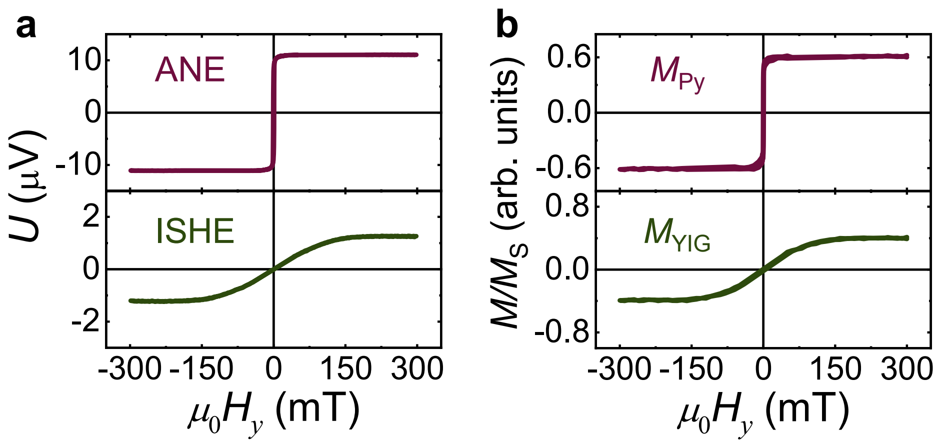

Supplementary Figure 5. The comparison of the deconvoluted ANE and ISHE contributions in the thermal voltage loop (**a**) and the magnetization contributions from Py and YIG in the magnetization loop (**b**), respectively. Apparently, they show close similarity, indicating the validity of the method for the deconvolution.

Supplementary Note 6. SOT-switching measurement of demagnetized Py

We measured the SOT-switching loops of PMA-YIG(10 nm)/Ag(4 nm)/Py(6 nm) both after demagnetizing Py, and with magnetic fields along the *x*-direction, shown in Supplementary Fig. 6. We find the SOT-switching cannot occur when Py is demagnetized, due to the absence of collinear spin current. In this sense, SOT-switching is similar in PMA-YIG/Ag/Py (demagnetized) and PMA-YIG/Pt with only conventional *y*-direction polarized spin current.

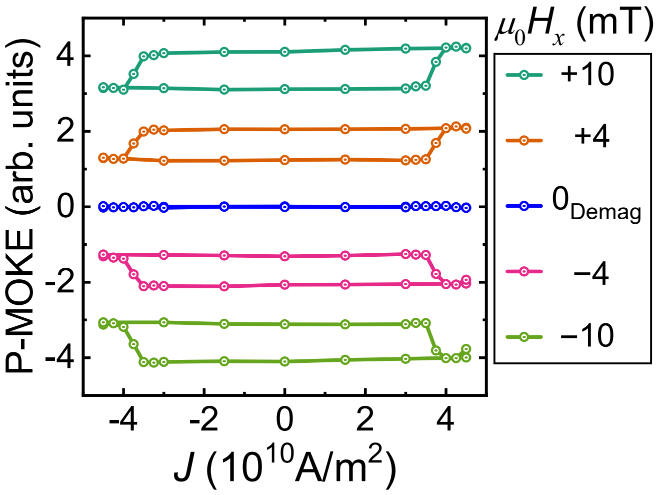

Supplementary Figure 6. SOT-switching loops of PMA-YIG(10 nm)/Ag(4 nm)/Py(6 nm) both after demagnetizing the Py layer and with magnetic field along *x*-direction.

Supplementary Note 7. The normalization procedure for P-MOKE measurement

In the MOKE measurement, a linearly polarized laser strikes on the sample, then reflects off the sample through a polarizer and into a photodetector. When the sample is magnetized, the polarization direction of the light rotates, then the voltage measured by the photodetector (proportional to the light intensity) changes. In the hysteresis loop measured by P-MOKE (scanning H_z), we first subtract the linear background caused by the Faraday effect in the object lens. Then the remaining voltage difference for positively and negatively saturated states comes from the PMA-YIG only. The normalization actually involves two process. We first subtract the Kerr signal with the mean value of the measured loop. Then further normalize the Kerr signal to the saturation value at the positive field. In this way, we obtain the out-of-plane hysteresis loop of PMA-YIG measured by P-MOKE (such as Fig. 3b in main text). For the SOT-switching loop measurements by P-MOKE (such as Figs. 3c and 3d in main text), the magnetic field is constant thus Faraday effect-induced signal (from the lenses) does not change. We thus can directly normalize the saturated voltage difference for positive and negative currents in SOT-switching loop to the saturated voltage difference for positive and negative fields in magnetic hysteresis loop. Because the current intensity does not involve in the normalization process, the meaning of the variation of current intensity will not change.

Supplementary Note 8. The coupling between Py and PMA-YIG

In this section, we discuss two types of coupling between Py and PMA-YIG, which are stray field in nano-pillar system and Néel orange-peel effect.

It was reported in nano-pillar system (dimension ~300 nm × 100 nm) that the stray field of an in-plane magnetized CoFeB could induce the field-free switching of perpendicular magnetized CoFeB[7]. However, our Py is patterned in a 100-μm long and 40-μm wide strip with two large pads at both ends (Fig. 3a in main text). In this situation, the generated stray field is expected to be very small when the magnetization is along the strip direction. In addition, the stray field is antiparallel with the magnetization direction of Py. The switching polarity from stray field is thus opposite to that from ASHE in Py observed in Figs. 3c&3d (in main text). Because the ASHE in Py is proportional to M_x of Py, we observed the correlation between the P-MOKE amplitude during SOT-switching and the L-MOKE (Py) behavior.

The Néel orange-peel effect was used to explain the field-free switching in the in-plane CoFeB/W/PMA-CoFeB sandwich structures[8-11]. In the SOT-switching measurement of PMA-YIG/Pt in Fig. 4e (in main text), we find the current density threshold almost does not change with the external magnetic field along x-direction within ±10 mT. The field-free SOT-switching achieved by magnetic coupling can be considered to be approximately equivalent to the conventional SOT-switching with the magnetic field applied along the charge current direction. As the spin Hall angle of Py is smaller than that of Pt, it is expected that in PMA-YIG/Ag/Py, the current density threshold for SOT-switching achieved by Néel orange-peel effect should be larger than

that in PMA-YIG/Pt, which is opposite to what we observed. According to previous theoretical prediction[12], the *z*-polarized collinear spin current is critical to reduce the SOT-switching current density threshold. Therefore, though the Néel orange-peel mechanism may also have its contribution, the reduced switching current density threshold as compared with that in PMA-YIG/Pt indicates that the field-free SOT-switching is mainly from the collinear spin current in Py.

References

1. Miao, B. F., Huang, S. Y., Qu, D. & Chien, C. L. Inverse spin Hall effect in a ferromagnetic metal. *Phys. Rev. Lett.* **111**, 066602 (2013).
2. Wang, H., Du, C., Chris Hammel, P. & Yang, F. Spin current and inverse spin Hall effect in ferromagnetic metals probed by $Y_3Fe_5O_{12}$-based spin pumping. *Appl. Phys. Lett.* **104**, 202405 (2014).
3. Du, C., Wang, H., Hammel, P. C. & Yang, F. $Y_3Fe_5O_{12}$ spin pumping for quantitative understanding of pure spin transport and spin Hall effect in a broad range of materials (invited). *J. Appl. Phys.* **117**, 172603 (2015).
4. Yang, W. L. et al. Determining spin-torque efficiency in ferromagnetic metals via spin-torque ferromagnetic resonance. *Phys. Rev. B* **101**, 064412 (2020).
5. Kimura, T., Hamrle, J. & Otani, Y. Estimation of spin-diffusion length from the magnitude of spin-current absorption: Multiterminal ferromagnetic/nonferromagnetic hybrid structures. *Phys. Rev. B* **72**, 014461 (2005).
6. Kimura, T. & Otani, Y. Large Spin Accumulation in a Permalloy-Silver Lateral Spin Valve. *Phys. Rev. Lett.* **99**, 196604 (2007).
7. Zhao, Z., Smith, A. K., Jamali, M. & Wang, J. P. External-Field-Free Spin Hall Switching of Perpendicular Magnetic Nanopillar with a Dipole-Coupled Composite Structure. *Adv. Electron. Mater.* **6**, 1901368 (2020).
8. Chen, W., Qian, L. & Xiao, G. Deterministic Current Induced Magnetic Switching Without External Field using Giant Spin Hall Effect of β-W. *Sci. Rep.* **8**, 8144 (2018).
9. Murray, N. et al. Field-free spin-orbit torque switching through domain wall motion. *Phys. Rev. B* **100**, 104441 (2019).
10. Yang, W. L. et al. Role of an in-plane ferromagnet in a T-type structure for field-free magnetization switching. *Appl. Phys. Lett.* **120**, 122402 (2022).
11. Kao, S.-C. et al. Field-free magnetization switching through modulation of zero-field spin-orbit torque efficacy. *APL Mater.* **11**, 111104 (2023).
12. Lee, D.-K. & Lee, K.-J. Spin-orbit Torque Switching of Perpendicular Magnetization in Ferromagnetic Trilayers. *Sci. Rep.* **10**, 1772 (2020).
