# Supplementary material for: Highly efficient field-free switching of perpendicular yttrium iron garnet with collinear spin current
Source: Nat Commun. 2024 Apr 13;15:3201. doi: 10.1038/s41467-024-47577-x (PMC11016059; doi:10.1038/s41467-024-47577-x)
Supplement: Supplementary file 3 — Reporting Summary [file 41467_2024_47577_MOESM3_ESM.pdf]

Corresponding author(s): Bingfeng Miao, Xiangrong Wang, Haifeng Ding

Lasing Reporting Summary

Nature Research wishes to improve the reproducibility of the work that we publish. This form is intended for publication with all accepted papers reporting claims of lasing and provides structure for consistency and transparency in reporting. Some list items might not apply to an individual manuscript, but all fields must be completed for clarity.

For further information on Nature Research policies, including our data availability policy, see Authors & Referees.

Experimental design

Please check: are the following details reported in the manuscript?

1. Threshold

   Plots of device output power versus pump power over a wide range of values indicating a clear threshold
   - [ ] Yes
   - [x] No

   In our MOKE measurements, we only used low power laser with a fixed power of 17 mW. We mentioned this in the methods.

2. Linewidth narrowing

   Plots of spectral power density for the emission at pump powers below, around, and above the lasing threshold, indicating a clear linewidth narrowing at threshold
   - [ ] Yes
   - [x] No

   It is not relevant to our research.

   Resolution of the spectrometer used to make spectral measurements
   - [ ] Yes
   - [x] No

   It is not relevant to our research.

3. Coherent emission

   Measurements of the coherence and/or polarization of the emission
   - [ ] Yes
   - [x] No

   It is not relevant to our research.

4. Beam spatial profile

   Image and/or measurement of the spatial shape and profile of the emission, showing a well-defined beam above threshold
   - [ ] Yes
   - [x] No

   It is not relevant to our research.

5. Operating conditions

   Description of the laser and pumping conditions
   Continuous-wave, pulsed, temperature of operation
   - [x] Yes
   - [ ] No

   In the Methods part.

   Threshold values provided as density values (e.g. W cm^{-2} or J cm^{-2}) taking into account the area of the device
   - [ ] Yes
   - [x] No

   It is not relevant to our research.

6. Alternative explanations

   Reasoning as to why alternative explanations have been ruled out as responsible for the emission characteristics
   e.g. amplified spontaneous, directional scattering; modification of fluorescence spectrum by the cavity
   - [ ] Yes
   - [x] No

   It is not relevant to our research.

7. Theoretical analysis

   Theoretical analysis that ensures that the experimental values measured are realistic and reasonable
   e.g. laser threshold, linewidth, cavity gain-loss, efficiency
   - [x] Yes
   - [ ] No

   Supplementary Note 7 in Supplementary Information.

8. Statistics

   Number of devices fabricated and tested
   - [ ] Yes
   - [x] No

   It is not relevant to our research.

   Statistical analysis of the device performance and lifetime (time to failure)
   - [ ] Yes
   - [x] No

   It is not relevant to our research.
